# Notes and News

**Published:** 1900-04-28

**Authors:** 


### Notes and News.

A cottage hospital has been opened at Colne, in Lancashire, by Lord Derby.

The Royal Hospital for Incurables, Putney, and the London Homœopathic Hospital each benefit to the extent of £500 by the will of the late Mr. J. J. Brown, of Reigate.

A new cottage hospital has been opened at Ramsbottom, near Manchester. The hospital is the gift of Mr. and Mrs. Aitkin, of Holcomb Hall. It is to be supported by voluntary contributions, andprovides for nine patients.

The outbreak of plague at Sydney, although dealt with energetically by Dr. Ashburton Thompson, the able chief medical officer of the New South Wales Government, has been one of considerable severity. Reuter's telegrams report that up to April 23rd 137 cases of plague had occurred, with 48 deaths.

Mr. Passmore Edwards, on becoming acquainted with the fact that the enlargement of the Passmore Edwards Cottage Hospital at Tilbury was necessary, has promised £500 towards the £1,000 necessary on condition that the public shall co-operate with him and provide the remaining £500. The East and West India Dock Company have given the land, and contributions in money will be received by Mr. A. Boxer, 50, Mark Lane, E.C.

The good people of Philadelphia are at last beginning to see some hope of obtaining filtered drinking water. The sand filtration plant at Albany seems to be recognised as a standing proof of the possibility of filtering water on the large scale, and Philadelphia is about to establish experimental works on the same plan, and if these should prove successful the whole of the water supply to the city will be dealt with by sand filtration. It is very curious to note the tentative manner in which the filtration of water on the large scale is being tackled in America, as if it were some new and startling innovation.

Professor Victor Horsley will deliver the second Lees and Raper Memorial lecture at St. James's Hall, Piccadilly, on Friday, April 27th, at eight p.m. The subject will be "The Effect of Alcohol on the Human Brain."

In consequence of the death of Sir William Priestley a vacancy occurs in the Parliamentary representation of the Universities of Edinburgh and St. Andrews. Sir John Batty Tuke, well known as a teacher and writer on mental diseases, has, we understand, consented to become a candidate. He is a Conservative, and thus his election would cause no change in the balance of parties in the House.

The Parliamentary paper issued last Thursday, giving particulars of the sight tests used in the mercantile marine during last year, is not altogether satisfactory reading. That there is a weak spot in the examination in colour vision seems clear from the considerable number of cases in which the decision first arrived at was revised on appeal. Of the 43 who failed in colour vision, 13 were re-examined on appeal, and of these four passed. According to this in nearly a third of the cases which appealed against their rejection for this cause an error had been made.

A consumption sanatorium for Westmoreland has been opened at Meathorp, near Grange. The building was originally a convalescent home, which has been altered to suit the present requirements. The administrative portion occupies the main block, and on the west side on the ground floor is the male ward, and above it the female ward. There are also two small wards for special cases. On the south side of the building are placed eight shelters. The patients will be drawn from the poorer classes in Westmoreland, and only those in the earlier stages will be received. When possible patients will be charged five shillings weekly towards their maintenance, but it is hoped to gradually endow, or obtain definite subscriptions towards the up-keep of free beds. Nine hundred pounds per annum has been promised towards the expense of the establishment as an experiment for five years by Dr. Paget-Tomlinson, who has himself equipped the building.

The greatest possible gratification will be felt throughout the medical profession at the announcement that the Queen has been graciously pleased to signify her intention to confer the decoration of the Victoria Cross upon Major William Babtie, C.M.G., of the Royal Army Medical Corps, for his conspicuous bravery at the battle of Colenso. The act of courage for which he was recommended for this distinction is thus described in the *Gazette*: "At Colenso, on the 15th December, 1899, the wounded of the 14th and 66th Batteries, Royal Field Artillery, were lying in an advanced donga close in the rear of the guns without any medical officer to attend to them, and when a message was sent back asking for assistance, Major W. Babtie, R.A.M.C., rode up under a heavy rifle fire, his pony being hit three times. When he arrived at the donga, where the wounded were lying in sheltered corners, he attended to them all, going from place to place exposed to the heavy rifle fire which greeted any one who showed himself. Later on in the day Major Babtie went out with Captain Congreve to bring in Lieutenant Roberts, who was lying wounded on the veldt. This also was under a heavy fire."

